# Reply to: Temporal lobe epilepsy and its negative impact on quality of life: Could it improve through aerobic physical exercise?

**DOI:** 10.1055/s-0046-1817032

**Published:** 2026-03-12

**Authors:** Shai O. Laks, Nathalia Volpato, Nikolas Coelho, Mateus Henrique Nogueira, Clarissa L. Yasuda, Luciana Ramalho Pimentel-Silva, Fernando Cendes

**Affiliations:** 1 Universidade Estadual de Campinas, Faculdade de Ciências Médicas, Departamento de Neurologia, Campinas SP, Brazil.

Dear Editor,

We would like to thank García Borja and Carranza for their interest in our study and welcome the opportunity to discuss key methodological aspects and findings.

In their letter, García Borja and Carranza state that our study intervention—a 6-month aerobic physical exercise (APE) program—is a “non-pharmacological treatment aimed at improving quality of life (QoL).” We want to clarify that we aimed to investigate whether a supervised APE program could enhance the QoL of people with temporal lobe epilepsy (TLE) and influence patients' concerns regarding seizures, while effectively improving aerobic fitness. Although closely related, “treatment” and “intervention” are distinct concepts in this context. This distinction is important as it defines which conclusions can be drawn from our study, given its scope and goals.

The study intervention was carried out in 2017; we appreciate García Borja and Carranza for pointing out this missing chronological detail. For context, we used the 2017 ILAE classification, 1 the standard in place until the recent 2025 updates. 2 Thus, the study's temporal context does not preclude valid interpretations of our findings.

Although our sample size is modest, we respectfully disagree with the claim that our non-significant results are solely due to insufficient statistical power, “making the findings less specific and conclusive.” Our study design allowed modelling within- and between-group interactions, which increases statistical rigor and minimizes type-I errors compared to multiple subgroup testing. 3 4 We demonstrated that physical capacity and overall QoL significantly improved in the intervention group only, despite no significant changes in the seizure worry domain. This indicates that the findings are related to the APE program and, therefore, specific to the intervention. Regarding the mention of “high *p* -values,” we assume the authors refer to the non-significant differences in clinical features. We maintain that a *p* -value should not be interpreted in terms of “strength” but rather against the established threshold of significance. To move beyond a *p* -value-only interpretation, we reported effect sizes, which showed moderate-to-large strength for significant outcomes, while non-significant differences had small effect sizes. These non-significant results across clinical characterizations likely indicate that the effects of potential confounders were negligible, reflecting our success in recruiting a representative sample. Therefore, we disagree that the influence of confounders cannot be appreciated, since the main clinical features that could influence outcomes were clearly described and statistically tested.

Our choice of tools stems from the use of gold-standard, validated methods: the cardiopulmonary exercise test for aerobic fitness 5 and the Quality of Life in Epilepsy Inventory (QOLIE-31) 6 for QoL. These tools are robust and sensitive for use in the epilepsy population in the context of physical activity, as previously demonstrated by our group. 7 Regarding blinding, in this study design, assignment blinding is neither a requirement nor feasible, as the control group does not undergo a sham intervention. Furthermore, the QOLIE-31 is a self-reported inventory, which inherently minimizes rater bias.

Finally, García Borja and Carranza raised valid safety concerns. While we agree that seizure control status should be taken into account, there is evidence of minimal or no influence of exercise in predisposing seizures in humans and animal models, 8 9 as discussed in our report. Nonetheless, our study was an exploratory pilot study on supervised APE and QoL, not a clinical trial designed to assess seizure risk and the safety and efficacy of physical exercise. Consequently, we could offer only insights into the safety of supervised aerobic exercise with individualized intensity.

In conclusion, we believe that our findings were appropriately interpreted within the context and boundaries of our research question. Our study indicates that supervised APE improved QoL without significant changes in seizure worry in a well-defined sample of people with chronic TLE. Supervised, systematic exercise could serve as a valuable tool in lessening the burden of TLE on wellbeing. Our findings warrant further investigation into other biopsychosocial aspects of epilepsy, and large-scale randomized trials remain necessary to determine the definitive safety and efficacy of exercise on seizure burden.
